# Design and development of a modified runway model of mouse drug self-administration

**DOI:** 10.1038/srep21944

**Published:** 2016-02-23

**Authors:** Vijayapandi Pandy, Yasmin Khan

**Affiliations:** 1 Department of Pharmacology, Faculty of Medicine, University of Malaya, 50603 Kuala Lumpur, Malaysia

## Abstract

The present study established a novel mouse model of a runway drug self-administration in our laboratory. The operant runway apparatus consisted of three long runways arranged in a zig-zag manner. The methodology consisted of six distinct phases: habituation, preconditioning, conditioning, post-conditioning, extinction and reinstatement. The effects of saline were compared with escalating doses of either ethanol (0.5–4.0 g/kg, i.p), heroin (5–40 mg/kg, i.p), or nicotine (0.1–0.5mg/kg, i.p) administered in the goal box during the conditioning phase (day 1 to day 5). A significant decrease in the time of trained (conditioned) mice to reach the goal box confirmed the subjects’ motivation to seek those drugs on day 6 (expression). The mice were then subjected to non-rewarded extinction trials for 5 days over which run times were significantly increased. After 5 days of abstinence, a priming dose of ethanol or heroin (1/5th of maximum dose used in conditioning) significantly reinstated the drug-seeking behavior. These results suggest that the modified runway model can serve as a powerful behavioral tool for the study of the behavioral and neurobiological bases of drug self-administration and, as such, is appropriate simple but powerful tool for investigating the drug-seeking behavior of laboratory mice.

Researchers have been using operant runways for the study of goal-seeking motivated behavior in rats for many decades^1^,^2^,^3^. Earlier reports demonstrated the self-administration of addictive drugs using a rat operant runway model^4^, but no such model exists for the study of drug-seeking in the mouse. In the current paper, we describe a modified runway paradigm that serves to lengthen the time required for the animal to reach the goal box, thereby limiting concerns about possible “floor effects” that can obscure group differences in animals (such as mice) that traverse the apparatus quickly. This is important since the primary dependent measure in such paradigms is the time required for the animal to travel across the length of the alley (i.e., Run Time) from the start box to the goal box where incentive drugs are given. Faster run times in runway models provide evidence of the animal’s motivation to seek the stimulus (such as a drug of abuse) that is made available upon goal box entry^4^,^5^.

The main drawback of the commonly used straight alley runway apparatus is an expression of very fast run times even in non-reinforced animals^5^. Therefore, the primary methodological changes were adopted in this current protocol with an aim of lengthening the alley by adjoining three runways in zig-zag manner that connected start and goal boxes. In addition, insertion of six small hurdles along three runways make the subjects to jump over and slowdown the speed of the animals to reach the goal box. This important strategical changes adopted in the present model can help the experimenter to differentiate drug-induced increases or decreases in operant behavior that can be more difficult to observe in the conventional straight alley approach.

It has been hypothesized that the dependence liability of addictive drugs are mediated by their activation of the endogenous neural systems normally engaged with natural incentives like water, food or sex^6^,^7^. This hypothesis inherently presume that addictive drugs represent a special class of positive reinforcers and, as such, can be studied utilizing the same operant/behavioral methods that are reliable to study the factors that play role in the initiation and maintenance of natural reward behaviors^4^.

The dopaminergic systems in the brain (particularly the mesocorticolimbic neurons, which originate from cell bodies in the ventral tegmental area and terminate in a number of brain regions include the amygdala, nucleus accumbens, and prefrontal cortex, play a significant role in goal seeking behavior of animals working for both natural and artificial reinforcers^8^,^9^. The goal-seeking behavior is directed by the activation of motivational and reinforcement processes in the brain^10^,^11^,^12^,^13^. The dopaminergic systems are inherently involved in both types of processes that control the goal-seeking behaviour^4^.

Ettenberg (2009) extensively reviewed the runway drug self-administration model and demonstrated that this paradigm was well-established in rats working for different addictive drugs such as intravenous (IV) opioid receptor agonists (morphine, heroin, remifentanil and alfentanil), IV cocaine, IV nicotine, IV “speedball” (heroin + cocaine), IV methylenedioxymethamphetamine (MDMA), subcutaneous (SC) morphine, SC amphetamine, and oral ethanol^4^. To date, however a mouse model of runway drug self-administration has not been developed and reported in the literature. Thus the present study aimed to establish a novel runway drug self-administration paradigm as a means of providing a simple but reliable method for the investigation of drug reinforcement in mice.

## Results

### Acquisition

**Figure 1:**
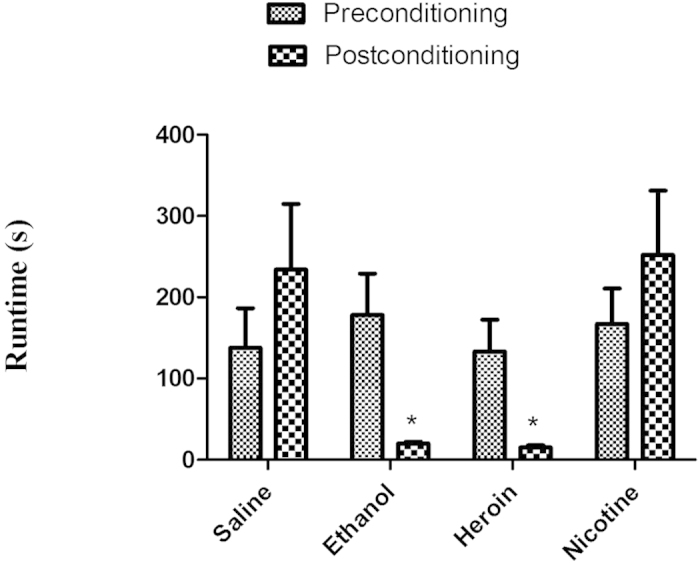
Acquisition of saline, ethanol, heroin and nicotine self-administration in mice- Mean (+/− SEM) run times during preconditioning and postconditioning of mice traversing a modified runway for access to different drugs delivered upon goal-box entry (n = 9/group). Asterisks* identify significant differences (*p* < 0.05) compared with the saline-control group.

Figure 1 depicts the pre- and post-conditioning run times of mice traversing the alley for access to different drugs delivered IP. A mixed two-factor (Group × Session) repeated measures ANOVA revealed a significant effect of drug treatment [*F* (3, 64) = 3.26; *p* = 0.0270] and a Group × Session interaction (*F* (3, 64) = 3.35; *p* = 0.0242). A separate one-way independent group ANOVA computed on just the preconditioning run times of the different treatment group (saline, ethanol, heroin and nicotine) was not statistically significant [F (3, 32) = 0.230, *p* = 0.566)]. On the other hand, the difference between groups (saline, ethanol, heroin and nicotine) on the post-conditioning run times were found to be statistically significant (F (3, 32) = 5.336, *p* = 0.0043). *Post hoc* analyses, Newman keuls revealed the ethanol- and heroin-treated groups, but not the nicotine group, to have significantly (*p* < 0.05) shorter run times when compared with the saline-treated control group.

### Extinction

**Figure 2:**
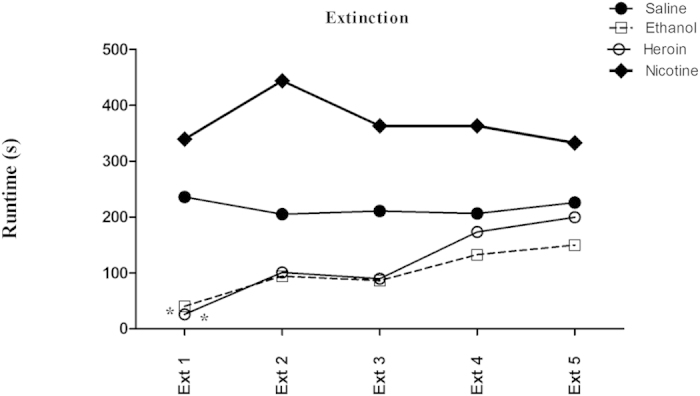
Extinction of saline, ethanol, heroin and nicotine self-administration in mice-The mean run time of each group of mice during Extinction Day 1 to Extinction Day 5 (n = 9/group). Asterisks depict a statistically significant difference (*p < 0.05) compared with the saline-control group.

Figure 2 depicts the run times of the saline, alcohol, heroin and nicotine groups upon removal of the treatments during the “extinction” phase of the experiment (Ext 1 to Ext 5) (Fig. 2). The mixed two-factor (Group × Session) repeated measures ANOVA revealed a significant effect of treatment [*F* (3,170) = 23.6; *p* = 0.0001] on run time. *Post hoc* analyses, Newman keuls revealed that ethanol and heroin significantly decreased the run time on Extinction Day 1 when compared with saline control.

### Reinstatement

**Figure 3:**
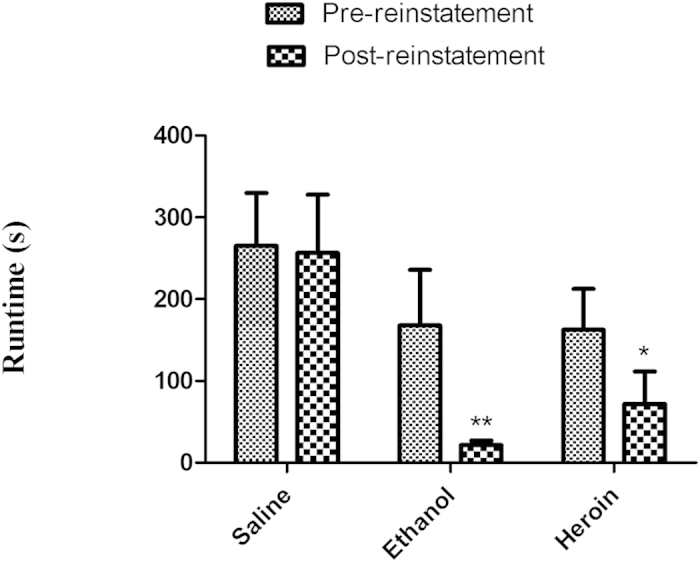
Reinstatement of saline, ethanol, heroin and nicotine self-administration in mice–Mean (+/− SEM) run times of the saline, ethanol and heroin groups on pre-reinstatement (Day 0) and post-reinstatement (Day 13) of testing. Significant differences reflect comparison with the saline-control group (*p < 0.05; **p < 0.01).

On Day 13, a priming dose of the each treatment was assessed for its ability to reinstate the runway behavior in each group of mice. These data are depicted in Fig. 3. A two-factor Group × Session ANOVA revealed a significant effect of Group [F (2, 46) = 5.35; *p* = 0.0082] but no significant effects for Session [F (1, 46) = 3.19; *p* = 0.0806] and no Group × Session interaction [F (2, 46) = 0.798; *p* = 0.457]. Separate one-way ANOVA results revealed no differences in the pre-reinstatement run times of the treatment groups (saline, ethanol and heroin) [F (2, 24) = 0.892, *p* = 0.423), but did confirm group differences in post-reinstatement run times [F (2, 24) = 6.89, *p* = 0.0047]. *Post hoc* analyses, Newman keuls revealed a significant reduction in post-reinstatement run time for both the ethanol (*p* < 0.01) and heroin (*p* < 0.05) groups when compared with the saline group.

**Figure 4:**
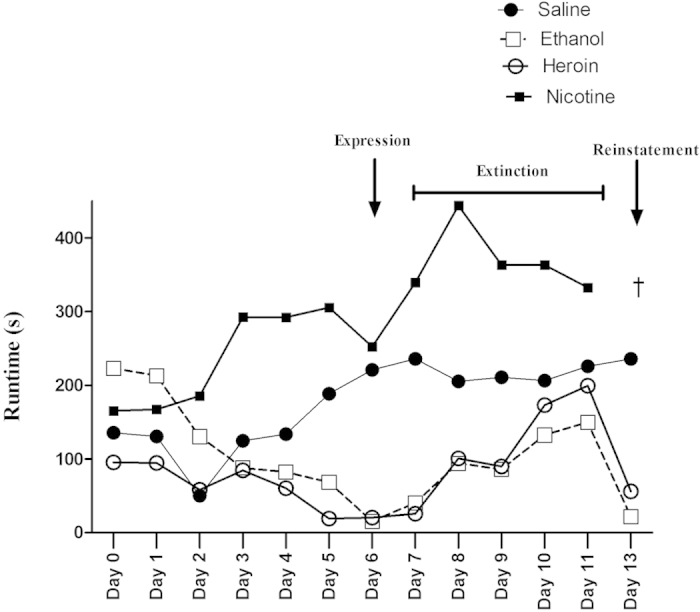
Mean run times for animals seeking saline, ethanol, heroin or nicotine during each phase of the experiment (from preconditioning to post-reinstatement). ^†^Note that the nicotine group was not tested for response reinstatement since this group did not exhibit faster run times for the drug during the initial acquisition phase of the experiment.

The overall performance from baseline (Day 0) to post-reinstatement (Day 13) of the different groups (saline, ethanol, heroin and nicotine) is shown in Fig. 4.

### Discussion/Conclusion

The escalating doses of ethanol and heroin from Day 1 to Day 6 significantly decreased run times, which indicated the positive reinforcing effects of ethanol and heroin. It has been observed that the run times began to decrease only after receiving two doses of ethanol and heroin, and reached a maximum after 5 doses of ethanol and heroin. These results are in good agreement with earlier published results using rats^14^,^15^. In contrast to these results, the escalating doses of nicotine (0.1–0.5 mg/kg, i.p) did not alter run times. This suggested that nicotine did not show any positive reinforcing effects at the selected escalating doses (0.1–0.5 mg/kg, i.p).

If indeed the reductions in run times during the initial phase of testing in the ethanol and heroin groups was a consequence of the drugs’ reinforcing effects, then learning theory would predict that removal of the reinforcer should produce extinction-like responding. This prediction was tested during the extinction phase of the study (Ext 1 to Ext 5) and indeed a gradual reduction in the drug-seeking behavior of the subjects in the ethanol and heroin groups was observed from the first to the last extinction trial. Once again, the response pattern for nicotine differed from the other drugs. Mice trained to run for nicotine exhibited no change in extinction behavior over trials. Moreover, the saline-control did not alter the running speed over the 5-days of extinction (Fig. 2). Thus, as is the case for rats, nicotine reinforcement is far more difficult to demonstrate than most other drugs of abuse. For example Tzschentke, 2007 extensively reviewed the place conditioning effect of various addictive drugs, including nicotine in rats and mice using a well-known conditioned place preference paradigm. Unlike other addictive drugs, nicotine showed highly variable results such in the conditioned place preference (CPP) test and often produced conditioned place aversion (CPA) at different dose regimens in different laboratories in different species and strains^16^. Most of the laboratories used subcutaneous as a route of administration for nicotine. In conclusion, the author suggested that nicotine can produce CPP only within a relatively narrow dose range and the exact range should be standardized in their own laboratories^16^. It has also been proposed that the rewarding effect of nicotine was highly influenced by the type of conditioning (“biased” or “unbiased”) method used in the CPP. The majority of studies that employed an unbiased conditioning procedure (where the drug is paired with both chambers of the apparatus in a counterbalanced manner) failed to produce nicotine CPP^17^,^18^. In contrast, studies using a biased procedure (where the drug is paired only with the initially less-preferred chamber of the apparatus) have reported success in developing a nicotine CPP^19^,^20^,^21^,^22^. When taken together, the most parsimonious explanation for these contrasting results is that nicotine might produce CPPs not via its rewarding/reinforcing attributes, but rather through an anxiolytic effect by reducing the aversive/negative aspects of the less preferred side^23^.

Similarly, it has proven to be relatively difficult to establish nicotine self-administration in rats despite its high dependence liability^24^. For example, some demonstrations of intravenous nicotine self-administration in rats was achieved only after introducing a response-contingent cue along with the nicotine infusions^25^,^26^. Light cues were commonly used to facilitate the acquisition of nicotine self-administration^26^,^27^,^28^,^29^,^30^. Unfortunately, the introduction of light cues in these studies was found to be more reinforcing than the nicotine itself^26^. Thus, overall acquisition of nicotine reinforcement *per se* cannot be optimized without co-presentation of response-contingent cues. Cohen and Ettenberg, (2007) also concluded that the intravenous injections of nicotine at doses above and below 0.03 mg/kg produced weaker drug-seeking behavior in a runway model and expressed relatively flat unchanged run speeds over days in rats^31^. Therefore, the failure to acquire nicotine self-administration in our newly developed mouse modified runway paradigm is not surprising because of nicotine’s relatively weak reinforcing properties^28^. Further studies using nicotine by changing the dose regimens and/or route of administration in the current runway paradigm are warranted. Studies in this direction are currently underway in our laboratory.

In short, the positive reinforcing effect of ethanol and heroin could be mediated through the direct activation of the reward pathways in the brain. Therefore, the current modified runway paradigm serves as a potentially important, yet simple, method for assessing the positive reinforcing effects of drugs of abuse. As such, it should prove to be a valuable tool for investigating the underlying neurobiological basis of drug addiction and abuse. Further studies are warranted to extend the current line of research to other addictive drugs in different phases of drug addiction such as acquisition, extinction and reinstatement.

## Methods

### Animals

Male ICR mice (UKM, Kuala Lumpur, Malaysia) weighing 25 to 30 g were housed in polycarbonate cages (n = 4/cage) for at least 7 days prior to the start of testing. All animals were maintained on a 12 h light/dark cycle (lights off at 7:00 pm) in, a temperature and humidity controlled vivarium (20 to 22 C, and a humidity of 45 to 60%). Animals were provided with free access to food pellets and purified drinking water. The animals were acclimatized to the housing unit and handled for 7 days before the start of the experimental session. Utmost care was taken to minimize the animal suffering. All experimental protocols were approved by the Institutional Animal Care and Use Committee, Faculty of Medicine, University of Malaya, Kuala Lumpur and care of the animals adhered to the guidelines of the National Research Council of the National Academies (“Guide for the Care and Use of Laboratory Animals”)^32^.

### Drugs

Ethanol (Copens Scientific, Malaysia), (−)-Nicotine hydrogen tartrate (Sigma-Aldrich, St. Louis, MO, USA), heroin hydrochloride (Chemistry Department, Ministry of Health, Malaysia) were used. Ethanol (10% v/v) was prepared by dilution of 95% v/v ethanol in sterile water for injection. (−)-Nicotine hydrogen tartrate and heroin hydrochloride solutions were prepared with normal saline. The pH of the nicotine solution was adjusted to 7.2 ± 0.2 using dilute NaOH solution. Freshly prepared drug solutions in normal saline were administered intraperitoneally (i.p) in a constant volume of 1 mL/100 g body weight of the animal.

### Modified Straight Alley Runway Apparatus

**Figure 5:**
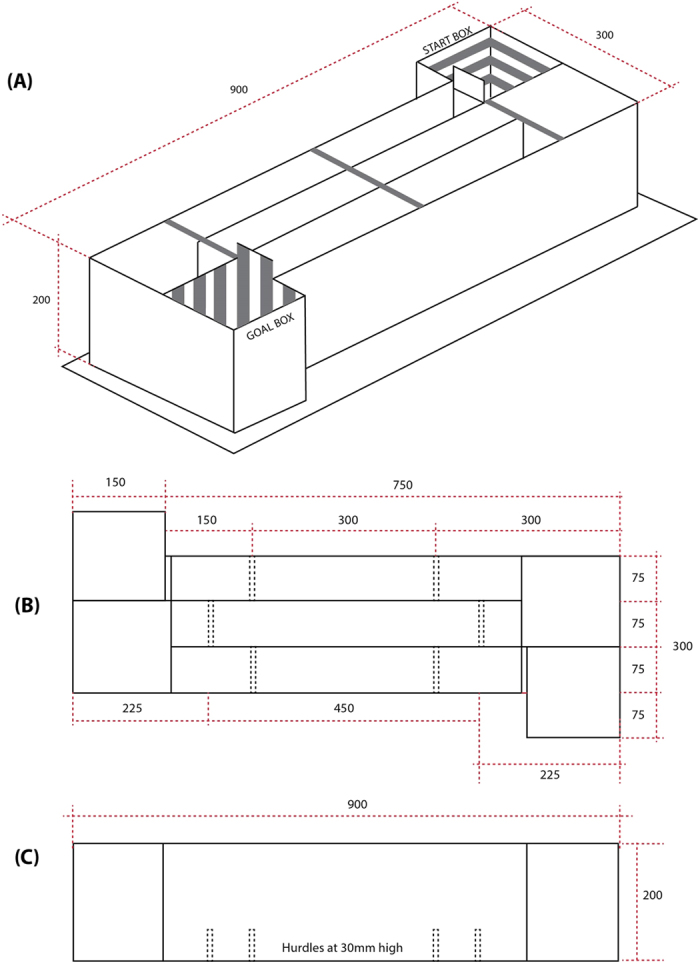
The schematic diagram of the modified straight alley runway apparatus (**A**) Top panel (**B**) Plan view (**C**) Side view. All measurements are shown in mm.

The runway apparatus was modified from earlier straight alley runway designs and incorporated a zig zag path to the goal box as a means of increasing the run times of mice traversing the apparatus (i.e., to prevent floor effects resulting from the relatively fast runtimes of the subjects). The apparatus was made of Composite Aluminium and arranged in a Z-shaped configuration as shown in the Fig. 5. This Z-shaped apparatus consisted of a square-shaped start box and a goal box each 150 mm (L) × 150 mm (W) × 200 mm (H). The start and goal boxes were connected with three straight runway segments (600 mm (L) × 75 mm (W) × 200 mm (H)) joined together in a zig-zag manner by two 150 mm curved segments (See Fig. 5A,B). The total runway distance from the start box to goal box was 1800 mm. The apparatus was situated on a tabletop at a height of 1200 mm from the floor (to minimize the mouse’s visual contact with the experimenter). Each segment of the runway included 2 hurdles at a height of 30 mm, to again reduce the speed with which the animals reached the goal box. The start box had black walls with white horizontal stripes and a black polished floor surface. A guillotine door separated the start box from the alley. In contrast, the goal box had white walls with black vertical stripes and a white wire-mesh floor. The location of the mice in the apparatus was recorded in real time using a Logitech webcam (C270) mounted above the apparatus and interfaced with a personal computer (PC) running custom software. The run time in seconds was recorded manually using a digital stop watch.

### Procedure

The testing protocol consisted of six distinct phases: habituation, pre-conditioning/baseline, conditioning/acquisition, post-conditioning, extinction and reinstatement. On the habituation day, each mouse was individually placed in the start box for 90 seconds, after which the guillotine door was lifted, thereby, allowing the animal to freely explore the straight alley portion of the runway (except the goal box) for 10 min. On the next day (Day 0), each mouse was allowed to run from the start box along the runway to the goal box. Immediately after goal-box entry the guillotine door of the goal box was closed to prevent retracing. The time interval between the start box door opening and the goal box door closing (run time in seconds) was recorded. This initial run time served as a baseline reading on the pre-conditioning day (Day 0). Immediately after recording the runtimes, the animals were returned to their home cages. Then the conditioning/acquisition phase was scheduled for next 5 days at 30-min daily conditioning sessions in the goal box (Day 1 to Day 5). During conditioning sessions, each mouse was allowed to run from the start box along the runway to the goal box with run times being recorded on each trial. Additionally, immediately after arrival of the animal in the goal box, the different treatment groups (saline/alcohol/heroin/nicotine; n = 9/group) received a single injection of the corresponding drug in escalating doses over a period of 5 days (Day 1 to Day 5). Subjects were then confined to the goal box for 30 min before being returned to their home cage. The saline group received one daily injection of saline (10 ml/kg, i.p.) from Day 1 to Day 5; the ethanol group received one daily injection of escalating doses of ethanol (0.5, 1, 2, 4 and 4 mg/kg, i.p.) from Day 1 to Day 5; the heroin group received one daily injection of escalating doses of heroin (5, 10, 20, 40 and 40 mg/kg, i.p.) from Day 1 to Day 5; and the nicotine group received one daily injection of escalating doses of nicotine (0.1, 0.2, 0.35, 0.5 and 0.5 mg/kg, i.p) from Day 1 to Day 5 of the conditioning phase. The post-conditioning (test day; Day 6) was scheduled 24 h after the last conditioning session. On post-conditioning day, the mice were placed in the start box for 90 seconds and then the guillotine door was lifted and the animals were allowed to move freely towards goal box. No drug or saline injections were provided on this “post-conditioning” trial. Starting 24-h hours after the post-conditioning test (i.e., from Day 7 to Day 11), mice were subjected for daily extinction testing. The run time was recorded daily during the extinction trials (Day 7 to Day 11). No injections were given during this extinction period. No treatment or testing was conducted on Day 12. One day later (Day 13), mice underwent reinstatement testing during which each subject received a priming dose of ethanol or heroin (1/5 th of maximum dose used in conditioning) prior to behavioral testing. The priming dose of ethanol and heroin was selected based on our preliminary studies and other published CPP studies^15^. Fifteen minutes after injection of the priming dose, the mice were individually placed in the start box for a single runway trial as described above for “post-conditioning”.

## Additional Information

**How to cite this article**: Pandy, V. and Khan, Y. Design and development of a modified runway model of mouse drug self-administration. *Sci. Rep.* **6**, 21944; doi: 10.1038/srep21944 (2016).
